# Effect of Omeprazole on Esophageal Microbiota in Dogs Detected Using a Minimally Invasive Sampling Method

**DOI:** 10.1111/jvim.70029

**Published:** 2025-02-26

**Authors:** Aditi Handa, Giovana S. Slanzon, Yoko M. Ambrosini, Jillian M. Haines

**Affiliations:** ^1^ Washington State University Pullman Washington USA; ^2^ University of Hawaii Manoa Hawaii USA

**Keywords:** dogs, esophagus, microbiome, omeprazole

## Abstract

**Background:**

Omeprazole alters the esophageal microbiome (EM) of humans and has associated effects.

**Objectives:**

To assess the changes and subsequent recovery of the EM in awake dogs after omeprazole treatment, using the esophageal string test (EST).

**Animals:**

Ten healthy, client‐owned adult dogs.

**Methods:**

A prospective longitudinal design was employed, where esophageal samples were initially collected using EST (day 0), involving the oral administration of an EST capsule and subsequent retrieval after 15 min for pH‐based segment identification. The dogs were then administered 1 mg/kg of omeprazole orally, twice daily for 14 days. Follow‐up EST samplings were conducted on days 15 and 45. Samples were sequenced targeting the V3‐V4 region of the 16S rRNA gene and diversity analysis along with differential sequencing (DEseq2) was performed.

**Results:**

All dogs tolerated the EST without adverse effects. The EST retrieved sufficient biofluid to characterize the EM in this group of dogs. Diversity analysis revealed no significant alterations in alpha (Observed species, Shannon and Simpson indices) and beta diversity (Bray‐Curtis) across the time points after omeprazole administration.

**Conclusions and Clinical Importance:**

Omeprazole therapy did not alter the EM of healthy dogs in this study. The application of EST in dogs illustrates its use as a minimally invasive tool for investigating the role of EM in esophageal health and disease in dogs.

**Abbreviations**
ASVs: amplicon sequencing variants
BE: Barrett's esophagus
DESeq2: Differential abundance analysis
EM: esophageal microbiome
EST: esophageal string test
GERD: gastroesophageal reflux disease
PPI: proton pump inhibitor

## 1 Introduction

The esophageal microbiome (EM) is a unique and under‐explored part of the host‐microbial ecosystem that plays a role in local health and homeostasis [1]. Niche microbiome investigations so far have identified alterations in microbial composition between normal and various esophageal disease states [1]. A landmark study in people suggests the classification of the EM into two groups: Type I microbiome dominated by Gram‐positive taxa in normal individuals and Type II microbiome dominated by Gram‐negative taxa in patients with gastroesophageal reflux disease (GERD) and Barrett's esophagus (BE) [2]. The majority of the EM studies describe phenomenological observations and associations rather than a definitive causal role [3]. Whether EM alteration induces esophageal disease remains unknown [4]. Although microbiota from various locations have been elucidated in veterinary medicine [5], the EM of dogs has not been researched in relation to esophageal health and common esophageal diseases, including gastrointestinal reflux disease, megaesophagus, and other motility disorders.

Proton pump inhibitors (PPIs) are a common class of medications used for the management of esophageal diseases in human and veterinary medicine. Gastric acid suppression with PPIs might impact the EM, and though the disease‐modifying effects and clinical implications of these changes are unknown, it has the potential to be quite relevant [6]. Examples of adverse effects reported with PPI use in people include PPI‐associated pneumonia and dysbiosis‐associated *Clostridioides difficile* infections [7, 8, 9]. Currently, there are no veterinary studies investigating the effects of PPIs on the EM, although omeprazole was found to alter the quantitative abundance of several bacterial communities in the gastrointestinal tract of dogs without any qualitative changes in the phylogenetic composition of the microbiota of the stomach and duodenum [10].

Previous studies investigating esophageal microbiota have utilized endoscopic mucosal biopsies or brush sampling techniques for sample collection. However, this is invasive, time‐consuming, and requires general anesthesia [11]. This complication risk under general anesthesia increases in patient groups that are already predisposed to reflux or regurgitation [12]. A minimally invasive capsule‐based string technology, called an “esophageal string test” (EST) has been used in awake human patients to sample the EM and the microbial profiles obtained were comparable to matched endoscopic biopsies [13]. This minimally invasive method could promote veterinary studies investigating the EM of dogs and help reduce the risk of complications associated with sampling in certain disease groups.

The aim of this study was to characterize the EM in a group of healthy dogs, evaluate the effect of short‐term omeprazole therapy (14 days) on the compositional change of the EM, and determine if the EM is restored by 30 days after the discontinuation of omeprazole. The study hypothesized that the EST would be safe and successful in obtaining esophageal biofluid for EM determination in healthy, unsedated dogs. We also hypothesized that short‐term omeprazole administered orally at a standard dose would cause changes to the EM composition, and restoration to the native state would be noted within 30 days.

## 2 Materials and Methods

### 2.1 Study Design and Dogs

This was a prospective longitudinal study design reviewed and approved by the institutional animal care and use committee at Washington State University. Clinically healthy, client‐owned dogs between the ages of 3 and 6 years were enrolled in the study. This age range was selected to avoid age‐related compositional changes to the microbiome [14]. A minimum weight of 9 kg was selected to avoid esophageal string‐associated complications related to body size. A physical examination, complete blood count, biochemical profile, urinalysis, and fecal examination were performed before enrollment to determine health status. A questionnaire was used to obtain information regarding their diet, environment, and medical history. Study dogs included had no previous history of gastrointestinal disease in the last 12 months and no gastro‐protectant, antimicrobial, or probiotic therapy for at least 3 months before enrollment [15]. Routine ectoparasite preventatives and joint supplements were allowed. A medication log was provided to the clients to document when they administered the medication to the study participants.

### 2.2 Sample Collection‐Esophageal String Test

Esophageal biofluid samples were collected by the esophageal string test (EST) method using the EnteroTracker (EnteroTrack LLC, Aurora, CO). The device comprises an ingestible capsule containing a weighted, stainless‐steel ball and a highly absorbent nylon string (90 cm, pediatric size). The kit provided by the commercial vendor (EnteroTrack LLC) contained a sterile thermoplastic sheet, a pair of scissors, forceps, measuring tape, pH indicator sticks (Figure S1A,B) and a colorimetric chart (Figure S2C). About 15–20 cm of string was pulled from the capsule until the beginning of the thicker absorbent string was visible. The capsule was orally administered by pilling, followed by 10–12 mL of water to ensure a complete swallow. The external portion of the string was secured to the dog's collar using an adhesive to allow free head movement. Using a permanent marker, a mark (0 cm) was made on the string at the level of the dog's lip/commissure. The string was allowed to remain in the dog's esophagus for 15 min (Figure S1E). The dogs were monitored for any discomfort and gently restrained to limit their movement in accordance with the principles outlined by the institutional ethics committee. After 15 min, the string was retrieved by pulling it out of the mouth at an even rate (over 2–3 s) per the manufacturer's recommendations (Figure S1F).

The string was placed on the sterile thermoplastic sheet for further processing. The 0 cm mark on the string was aligned with 0 cm on the tape measure. The distance from the oral commissure (0 cm) to the caudal aspect of the base of the ear was measured for each dog, and a second mark (~7–8 cm) was made on the string to avoid the pharyngeal portion. Per the manufacturer's recommendation, the pH indicator sticks were used to determine the esophageal portion of the string and distinguish gastric contents by blotting the string at 2 cm intervals and comparing it against the colorimetric chart (Figure S2A–D). To harvest the esophageal section of the string, the beginning was marked about 7–8 cm from the 0 cm mark, and the end was marked 2 cm from the beginning of the gastric section (pH < 4), if obtained, or from the end of the string. The esophageal section of the string was cut using the forceps and scissors in the kit provided. The esophageal section of the string was placed in the nucleic acid stabilizing solution (DNA/RNA shield, Zymo Research, Irvine, CA) and the vial was inverted 7–8 times after securing the lid to ensure complete submergence of the string (Figure S2D). The esophageal string samples were frozen within 4 h of collection and kept at −80°C until analysis.

### 2.3 Study Description

Using the method described, esophageal biofluid samples were collected using the EnteroTracker on day 0 (baseline) for all 10 dogs. The dogs were then orally administered a dose of 1 mg/kg of omeprazole, twice daily for 14 days (uncompounded omeprazole delayed‐release capsules, 10 mg and 20 mg strength, Dr. Reddy's, Telangana, India). A dosing chart was used for the study participants for consistent omeprazole dosing and to record any adverse effects. Follow‐up EST samplings were conducted on day 15 and day 45 using the method described for all 10 dogs. A plain string sample from a new EST kit was collected in the same manner described to minimize technical variation and was stored in the nucleic acid stabilizing solution as a negative control (Figure S3).

### 2.4 Microbiome Evaluation‐16 s rRNA Sequencing and Analysis

The stored samples contained in the nucleic acid stabilizing solution were thawed and sent to Zymo Research (Irvine, CA) for DNA extraction and 16S‐rRNA V3–V4 region amplification and sequencing. DNA extraction was performed using the ZymoBIOMICS‐96 MagBead DNA Kit (Zymo Research, Irvine, CA). The DNA samples were prepared for targeted sequencing with the Quick‐16S Plus Next Generation Sequencing (NGS) Library Prep Kit (Zymo Research, Irvine, CA). Primers were custom designed by Zymo Research to target the V3 – region of the 16S rRNA gene (Zymo Research, Irvine, CA). The final library was sequenced on Illumina NextSeq 2000 (600 cycles). Unique amplicon sequences were inferred from raw reads using Dada2 for bioinformatic analysis [16]. Chimeric sequences were also removed with the Dada2 pipeline. Taxonomy assignment was performed using Uclust from Qiime v.1.9.1. Taxonomy was assigned using the Zymo Research Database as a reference. Quantitative microbial data was estimated by means of a real‐time qPCR assay using a commercially available quantitative PCR assay kit (Quick 16 S qPCR Premix; Zymo Research Corp) and primers targeting the V3–V4 region. A commercial positive control was used as a standard (ZymoBIOMICS Microbial community DNA standard). The assay protocol consisted of an initial denaturation step at 95°C for 10 min; 20 cycles of denaturation at 95°C for 30 s, annealing at 55°C for 30 s, and extension at 72°C for 3 min; and a final hold at 4°C.

### 2.5 Statistical Analysis

A total of 10 dogs were determined to be appropriate to detect statistically significant changes in the microbiome, based on a priori power analysis [10].

Alpha diversity to estimate sample richness was calculated using the *vegan* package in R program (R package version 2.0‐10.2013) using the observed species, Shannon, and Simpson index metrics. Differences between alpha diversity indexes across groups were calculated by a non‐parametric approach using Kruskal‐Wallis test from R's package *stats*. Beta diversity analysis, which is the measure of compositional overlap between microbial communities, was calculated using the ordinate function in R's *phyloseq* package and represented as a principal coordinates analysis (PCoA) based on the Bray–Curtis dissimilarity [17]. The distances between the points in the principal coordinate space reflects the differences between communities. A permutational multivariate ANOVA (PERMANOVA) test was implemented with the *vegan* package in R. In this analysis, the strata argument within the adonis2 function of the R *vegan* package was used to control each dog's individual microbiome composition and assess its changes over time (r‐project.org/CRAN/refmans/vegan/html/adonis.html). Relative abundance data was calculated based on the number of sequence reads and total reads per sample and plotted using the ggplot2 R package (version 3.5.1). Absolute abundance was inferred using qPCR results. ANOVA and Kruskal‐Wallis tests were performed to assess differences across groups. To detect differential abundance of individual amplicon sequence variants (ASVs) between the groups (measured in log2 fold change), we used the package DESeq2 to compare day 0 versus day 15, day 15 versus day 45, and day 0 versus day 45 in consideration of the compositional nature of the data [18]. For all tests, *p* < 0.05 was considered significant.

## 3 Results

### 3.1 Study Group

A total of 10 dogs were enrolled in the study, ranging from 3 to 6 years of age. Mostly medium to large breeds were enrolled, including 2 Labrador Retrievers, 2 Great Pyrenees, 1 Border Collie, 1 Alaskan Malamute mix, 1 German Shepherd mix, 1 Bernese Mountain dog, 1 Pembroke Welsh Corgi, and 1 Cardigan Welsh Corgi. Their body weights ranged from 12.5 to 52 kg. All dogs were on a commercial diet. Sample collection using the esophageal string test was well tolerated by all the dogs. No adverse events were noted during sample collection. All dogs tolerated the course of omeprazole well, except one dog developed vomiting toward the end of the course, which resolved after omeprazole was administered with a small amount of food.

### 3.2 Esophageal Microbiome Analyses

**FIGURE 1:**
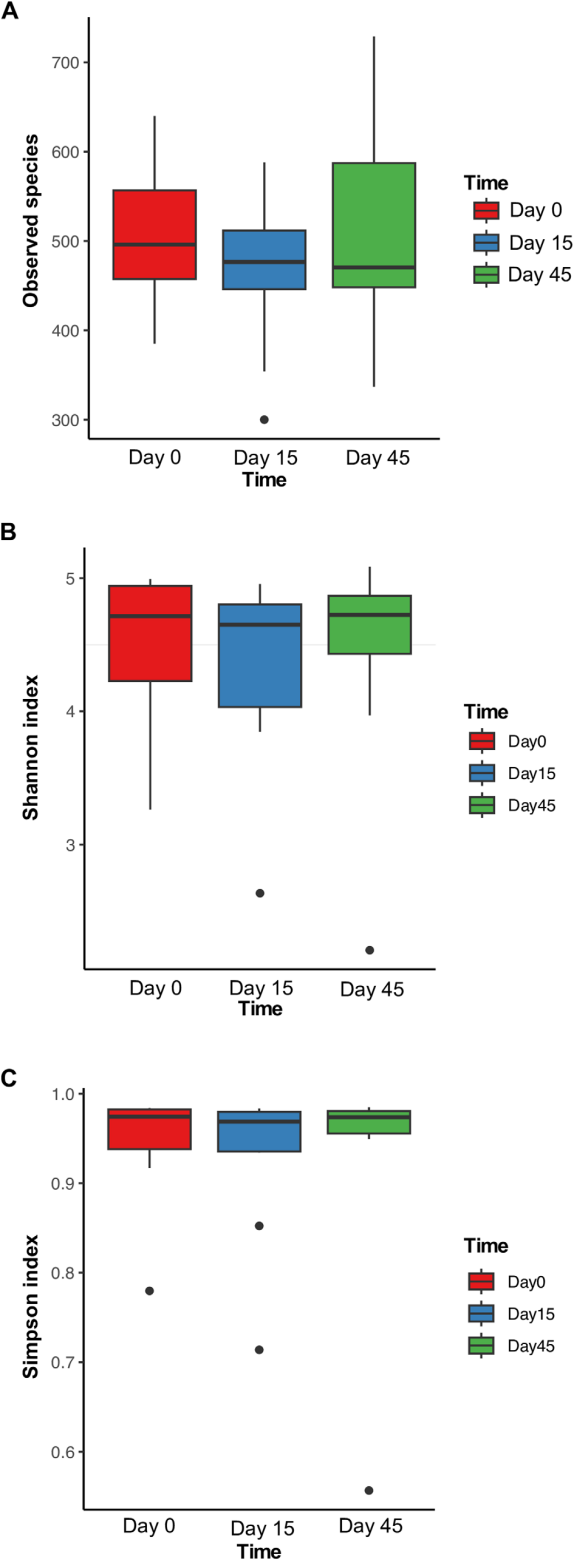
EM alpha diversity characterized by (A) Observed species index, (B) Shannon index, and (C) Simpson index of 10 healthy dogs at day 0, day 15, and day 45 of microbiome sampling. There were no significant differences among groups (Observed species, *p* = 0.717, Shannon index, *p* = 0.642, and Simpson index, *p* = 0.817).

A total of 2505 unique ASVs were identified across 30 esophageal biofluid samples, with 209602–329224 sequences per sample. Of those, 155 were shared between all dogs across all the time points. Microbiome diversity was compared using alpha and beta diversity indices. Alpha diversity measures the number and abundance of taxa in a local habitat. All three alpha diversity indices showed similar results across the time points in the study (day 0, 15, and 45) with no significant differences in EM over time (Observed species, *p* = 0.717, Shannon index, *p* = 0.642, and Simpson index, *p* = 0.817; Figure 1).

**FIGURE 2:**
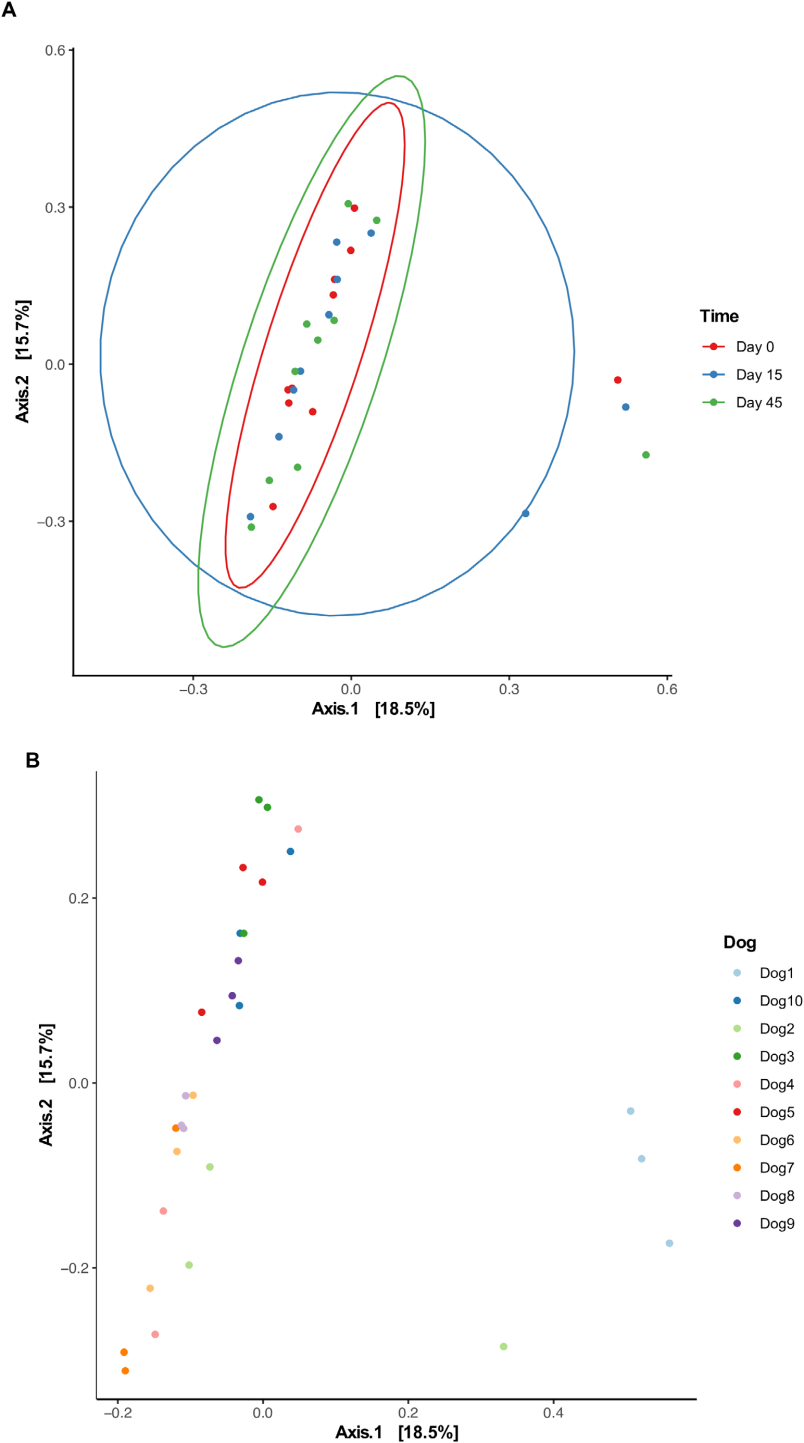
EM beta diversity of 10 healthy dogs using Bray Curtis dissimilarity grouped by (A) time and by (B) dog represented by principal coordinate analysis (PCoA) plot. We observed no significant difference within the EM of individual dogs over time while receiving omeprazole treatment (*R* ^2^ = 0.03 and *p* = 0.474. The variance in the samples is a function of the differences of the individual composition of each dog's microbiome which could be the main driver of the changes observed across samples (*R* ^2^ = 0.68 and *p* = 0.001).

Beta diversity, used to compare community composition across groups, was calculated using Bray–Curtis dissimilarity. Based on PERMANOVA results, we did not observe significant differences in the EM composition of samples collected from the same dog at different time points. Although a great proportion of the variance (*R* ^2^, coefficient of variance) of the data were explained by the difference across the individual composition of each dog's EM (*R* ^2^ = 0.68 and *p* = 0.001), we observed no significant difference within the EM of individual dogs over time while receiving the omeprazole treatment (*R* ^2^ = 0.03 and *p* = 0.474). These results suggest that each dog's unique microbiome was the main driver of the changes observed across samples (Figure 2).

**FIGURE 3:**
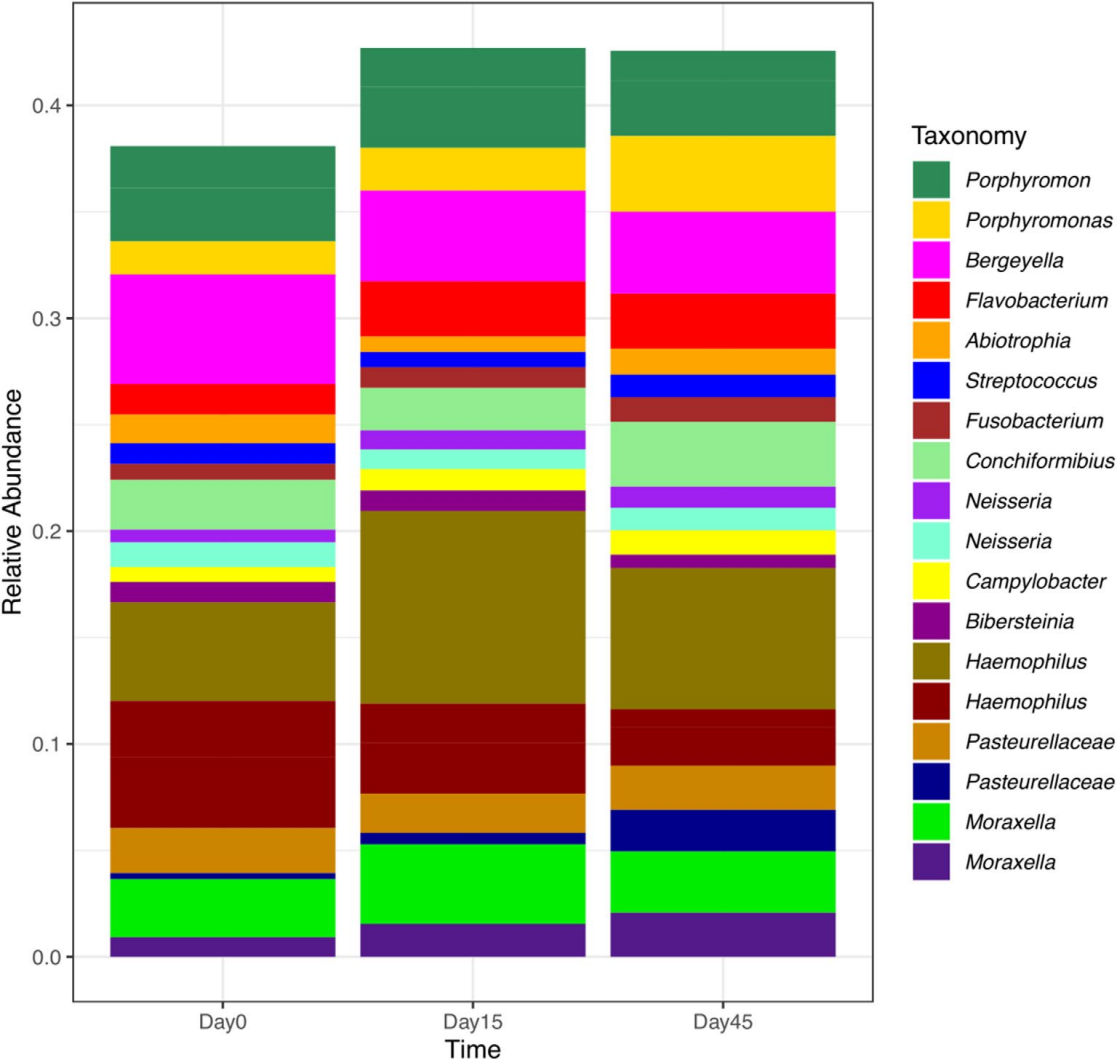
Bar plot showing mean relative abundance of EM of 10 healthy dogs on day 0, day 15, and day 45 of sampling. Bacterial amplicon sequencing variants (ASVs) were grouped by treatment period and annotated to the taxonomic level of genus. There is enrichment of certain genera across the time points but overall similar bacterial abundances after omeprazole therapy.

The ASVs were further classified according to genus, and their proportions were represented using a bar plot with their respective relative abundances compared across time (Figure 3 and Table S1). While there is a slight change in the proportion of distribution, the overall relative abundance of EM at the genus level does not change with omeprazole administration over the time points. The most common genera noted across these samples included *Fusobacterium, Bergeyella, Hemophilus, Porphyromonas, Flavobacterium, Streptococcus, Neisseria, Campylobacter, Moraxella, Bibersteinia, Conchiformibius, Abiotrophia*, and others from the family Pasteurellaceae.

**FIGURE 4:**
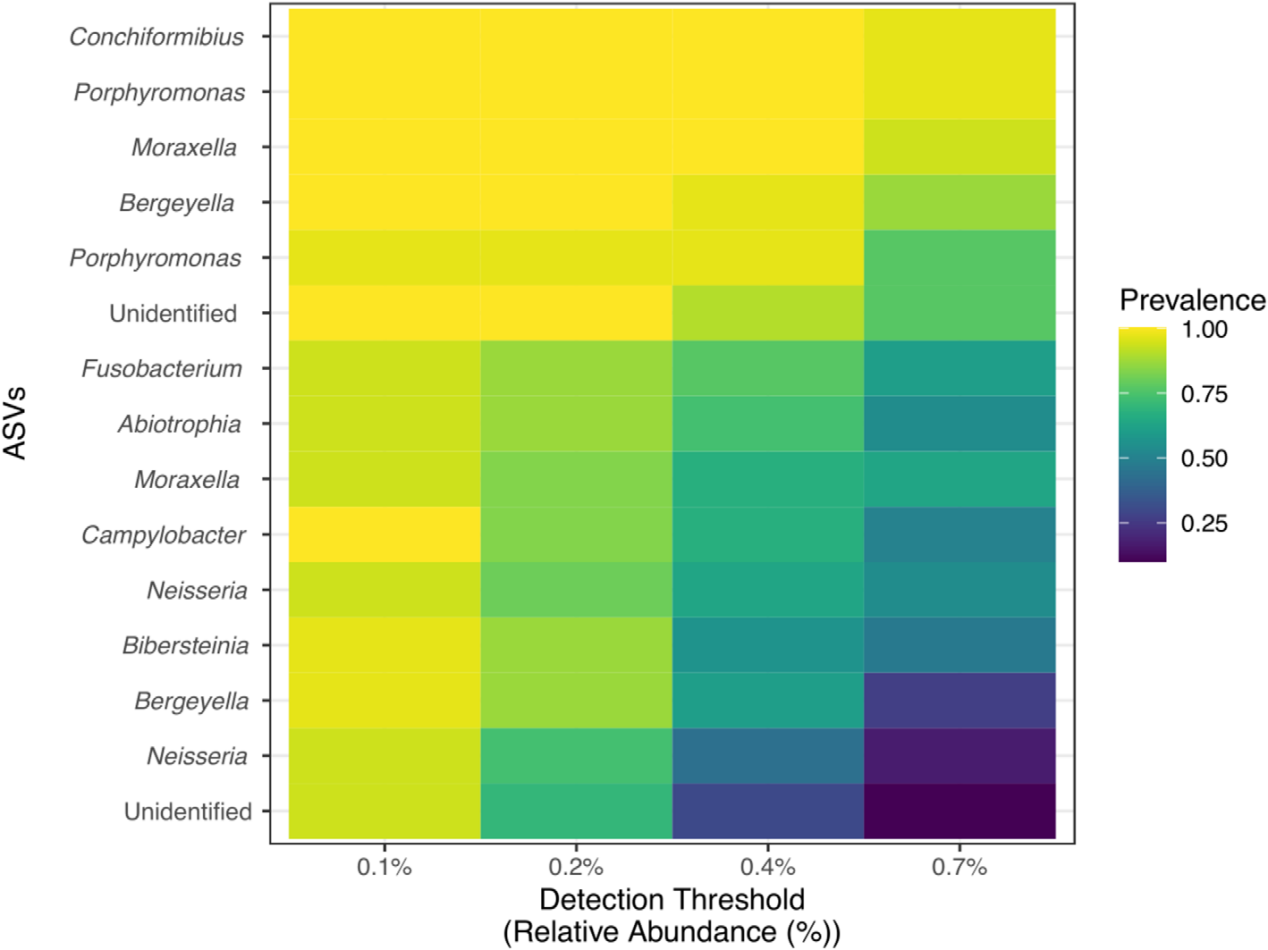
This figure represents a heat map displaying the 18 ASVs with higher prevalence and detection rates across all samples.

A core EM analysis across all samples was performed using cut‐offs including ASVs that were present in at least 50% of samples with a relative abundance greater than 0.01% [19]. A total of 311 ASVs were present in at least 50% of samples across all time points. However, only 6 of them were present in at least 50% of samples with a relative abundance > 0.01%. These include *Bergeyella zoohelcum* (sequence 4), *Moraxella cuniculi* (sequence 8), *Porphyromonas canigingivalis* (sequence 10 and 24), *Conchiformibius steedae* (sequence 12) Pasteurellaceae NA sp. 62273 (sequence 20), and *Moraxella sp62638* (sequence 30). Figure 4 highlights the 18 ASVs with higher detection and prevalence rates across the study's dataset. We have also included a table containing the taxa present in at least 50% of the samples (Table S2).

**FIGURE 5:**
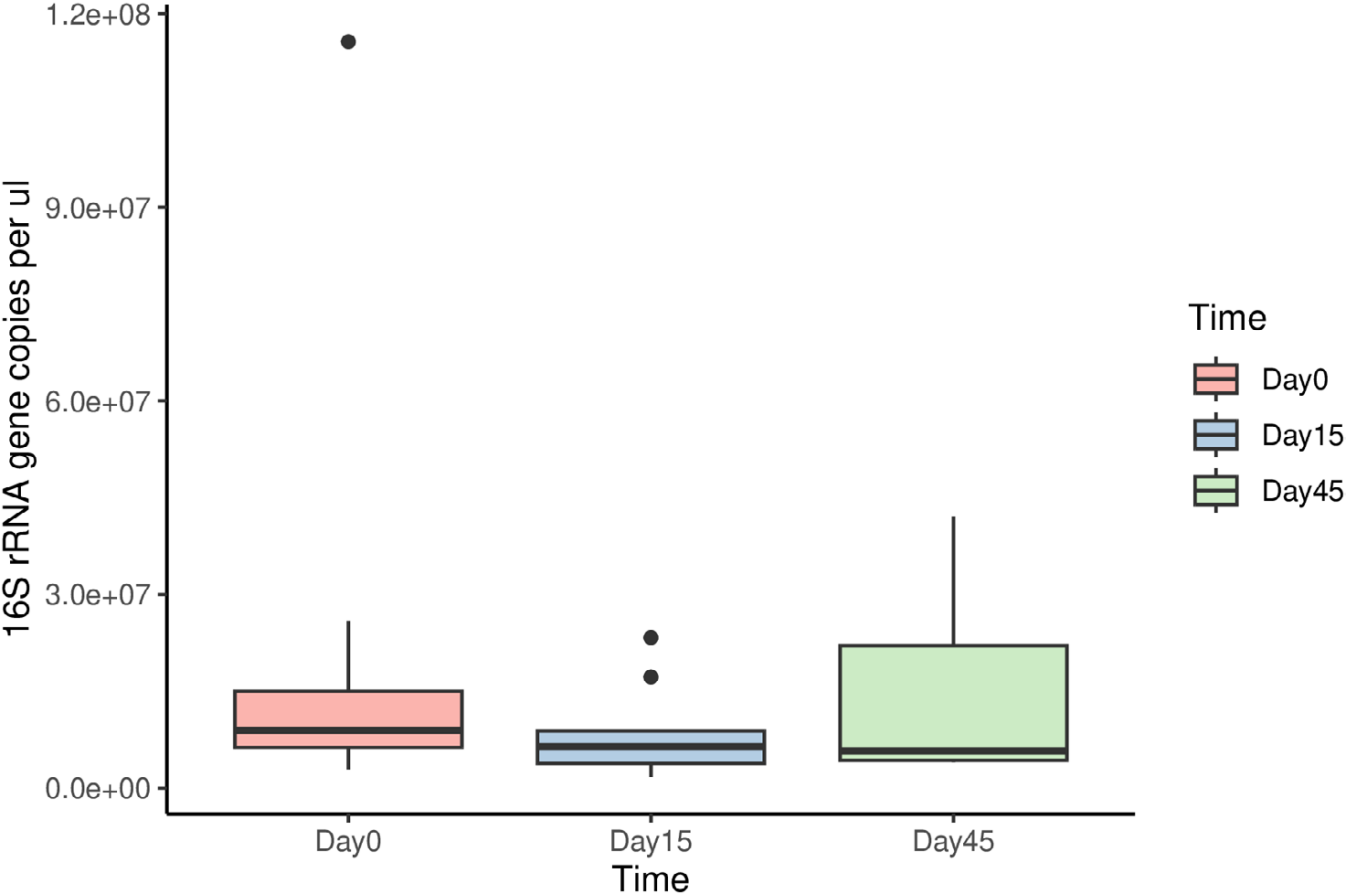
This box and whisker plot represents absolute abundance obtained from qPCR analysis. The *x*‐axis represents the time period of the study (day 0, day 15, and day 45) and the *y*‐axis represents the mean gene copies of the targeted gene per μL. No significant differences were observed across groups using ANOVA (*p* = 0.457) or Kruskal‐Wallis test (*p* = 0.565).

Results from the qPCR analysis were represented as absolute abundance (Figure 5) across the time periods. We did not observe differences across groups using ANOVA (*p* = 0.457) or Kruskal‐Wallis test (*p* = 0.565).

### 3.3 DEseq2 Analysis for Individual Comparisons Across Time

**FIGURE 6:**
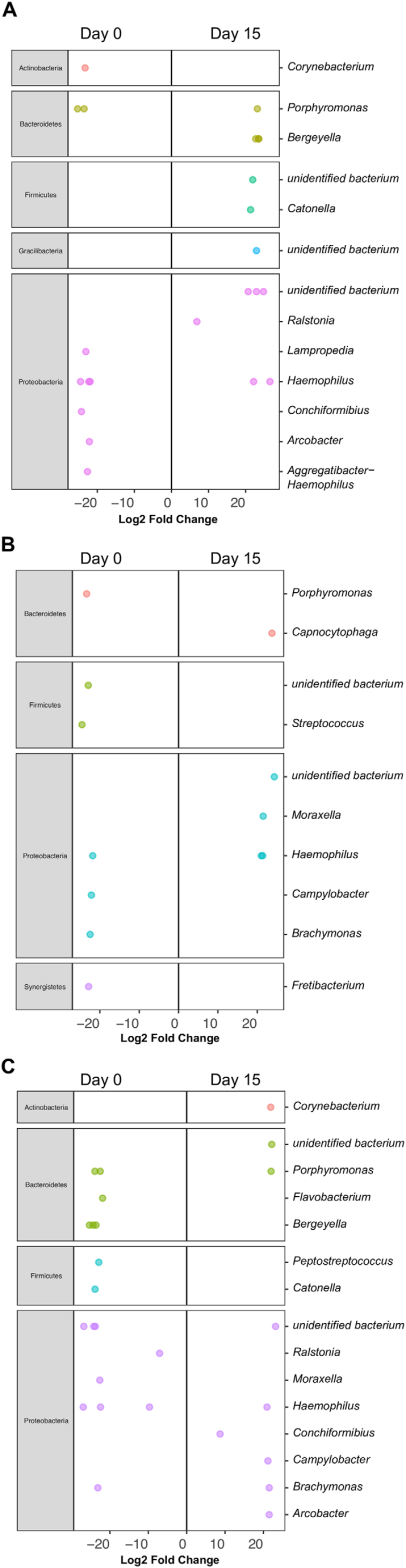
Differential abundance analysis (DESeq2) for comparison between groups. The significantly abundant bacterial ASVs across groups: (A) Day 0 versus Day 15, (B) Day 15 versus Day 45, (C) Day 0 versus Day 45. Each dot represents a single ASV, colored and grouped into a genus on each line.

Differential abundance analysis based on generalized linear regression model using negative binomial distribution‐ called DEseq2 was used for individual comparisons between day 0, day 15 and day 45. Figure 6 represents the significant differences of bacterial ASVs at the genus level between the groups when compared to each other. Based on the DEseq2 analysis it was observed that only a few taxa were differently enriched across the time points; however, the abundance changes were not sufficiently large to shift the community structure.

#### 3.3.1 Comparison Between Day 0 and Day 15

The taxa that were significantly enriched on day 15 include *Catonella, Bergeyella, and* an unidentified bacterium (Phylum‐Proteobacteria, Firmicutes). Similarly, there is a reduction in the abundance of certain taxa on day 15 compared to day 0, which includes *Lampropedia, Corynebacterium*, and *Porphyromonas*.

#### 3.3.2 Comparison Between Day 15 and Day 45

The taxa that were significantly enriched on day 45 include *Capnocytophaga, Moraxella, Hemophilus*, and unidentified bacterium (Phylum‐ Proteobacteria). Similarly, there is a reduction in the abundance of certain taxa on day 15 compared to day 45, which includes *Porphyromonas, Streptococcus, Campylobacter*, and *Brachymonas*.

#### 3.3.3 Comparison Between Day 0 and Day 45

The taxa that were significantly enriched on day 45 included *Corynebacterium, Porphyromonas, Campylobacter, Brachymonas*, and *Arcobacter*. Similarly, there is a reduction in the abundance of certain taxa on day 45 compared to day 0, which includes *Flavobacterium, Bergeyella, Peptostreptococcus, Catonella, Ralstonia, and Moraxella*.

## 4 Discussion

This study profiles the EM in dogs and demonstrates the effectiveness of the minimally invasive EST method for collecting esophageal biofluid. No significant differences in alpha or beta diversity or quantitative abundance were detected in the EM after short‐term omeprazole administration in healthy client‐owned dogs. The EST provides a safe and promising method for examining EM alterations in dogs with esophageal conditions or those at higher risk for aspiration or anesthesia‐related complications.

Compared to more invasive methods of esophageal sampling such as brush cytology and endoscopy, the EST offers a practical and effective alternative for EM sampling, omitting the need for general anesthesia [13]. With the growing body of knowledge regarding the esophageal microbiome and its alterations in disease states in human medicine [4], a test such as the EST could fuel further research of the EM, where repeated sampling would be advantageous to assess the stability of the microbiome or its response to interventions [13]. General anesthesia itself is a risk factor for gastro‐esophageal reflux, leading to consequences of esophagitis and esophageal strictures in veterinary patients [20]. In addition, dogs with esophageal dysmotility or megaesophagus are not ideal anesthetic candidates due to their increased risk of aspiration pneumonia [21, 22]. Brachycephalic dogs are prone to gastroesophageal reflux and due to their anatomical conformation, have a higher risk of complications when undergoing general anesthesia [23]. To encourage further microbiome studies in these disease groups, the EST allows for sampling with reduced risk and discomfort. In the present study, the EST was well tolerated with no adverse effects and allowed sufficient sampling of the esophageal biofluid in unsedated dogs. Bacterial profiles obtained through EST are equivalent to those obtained via endoscopic biopsies [13]. In addition, luminal sampling using the EST interrogates a larger surface area of the esophagus versus mucosal biopsies, thus allowing for a more comprehensive overview of EM [13].

Analysis of the esophageal mucosal microbiota of American individuals revealed that the most abundant genera were *Tissierella, Lactobacillus, Streptococcus, Acinetobacter*, and *Prevotella* [24]. Other studies exploring the EM in humans reveal an overall predominance of Gram‐positive bacteria, with *Streptococcus* as a consistently, highly abundant genus [1, 2, 3, 10]. This differs from the results of the EM samples of dogs in our study, where *Streptococcus* was not the most predominant genus, suggesting inherent species differences within the EM (Figure 3). Approximately 6 genera were found to be consistent across all samples and time points, suggesting their temporal stability (Figure 4). Further studies would be needed to characterize a core EM in dogs.

The use of PPIs has increased over the past decade, piquing research interest in potential adverse outcomes. As PPIs can influence the EM through pH‐dependent and pH‐independent factors [6], studies have explored the possibility of microbiome‐driven adverse effects such as PPI‐associated pneumonia, which are relevant to aerodigestive diseases [9]. Only a small drop in species richness was noted in subjects with a normal esophagus on PPIs in comparison to controls in another study, which concluded that PPI usage did not have a significant impact on alpha diversity measures or on the global taxonomic composition of the esophageal microbiota [25]. However, a study comparing the EM from esophageal biopsy samples after 8 weeks of PPI therapy in patients with esophagitis/Barrett's esophagus and controls revealed significant differences in the microbial communities after PPI therapy [2]. One study in human medicine documented an increase in the abundance of Firmicutes and a decrease in the abundance of Bacteroidetes and Proteobacteria in the esophagus after PPI therapy [7]. However, another study comparing PPI users and non‐PPI users revealed no significant difference in the EM, except for an increase in *Actinomyces spp*. The same study also concluded that neither dose nor duration of PPI had any effect on the microbial community of the distal esophagus [26].

This study explored the effect of short‐term omeprazole on the EM of healthy dogs. Similar to some previous human studies, this study found no significant differences in the alpha and beta diversity of the EM across the time points, suggesting no alteration to the EM after omeprazole therapy. Absolute abundance measured by qPCR provides a quantitative measurement of bacterial genes, which also confirmed the lack of changes to the EM after 2 weeks of omeprazole therapy. While this is an investigative study, the lack of alteration was unable to prove microbiome‐associated risk for adverse effects in this cohort. Additional studies are needed in veterinary medicine to corroborate risks of PPI use in veterinary medicine, and rational use is encouraged for correct indications and to avoid overuse [27].

Although there is divided evidence on the influence of PPIs on the EM, and lack of definitive association with pathology, on differential abundance analysis (DEseq2, Figure 6) an increase in certain genera (i.e., *Catonella*) contained within the families of Lachnospiraceae and Clostridales, and a reduction in Comamonadaceae family (i.e., *Lampropedia*) was seen on day 15 after PPI therapy. A human study by Amir et al. similarly found a reduction in the family Comamonadaceae and increases in families of Clostridiaceae, Lachnospiraceae, Microccocaceae and Actinomycetaceae following PPI treatment [28]. While there was a shift in some taxa that were significantly different, the overall community remained stable. The relevance of this change is unknown but could still indicate some effect on EM in dogs treated with PPIs.

There were some limitations to this study. This study looked at a diverse, healthy dog cohort, and while the population contained various breeds, age extremes were avoided to offset any age‐related influences [14]. Most dogs in the study were on a standard commercial diet, so dietary influence was unable to be studied [29].

Typically, sampling times with the EST are up to an hour in humans [13]. The duration of string placement selected was 15 min to allow sufficient sampling time with minimal discomfort, given the investigative nature of the study (Figure S4). More recently, shorter durations (15 min) appear to be sufficient for esophageal sampling in humans as well (data to be published). In the present study, we utilized pH‐based identification of the esophageal segment as recommended by the manufacturer. Future studies can include imaging with radio‐opaque markers on the string or endoscopy to assess the exact location of the string within the esophagus. Oropharyngeal contamination is not seen with the EST in humans [13]. While this approach has not been previously tested in dogs, further studies comparing oropharyngeal microbiota could help confirm its effectiveness in ensuring sample purity in our EST procedure.

Our study found no significant changes in the esophageal microbiome (EM) after 2 weeks of PPI administration. This duration has previously been shown to cause alterations in the gastric and fecal microbiomes of dogs [10] and was therefore expected to be sufficient for detecting changes in the EM as well. However, no such differences were observed. It is possible that the short course of omeprazole used in this study was insufficient to elicit a detectable treatment effect on the EM. Since PPI efficacy depends on binding to active H + ‐K + ‐ATPase enzymes, plasma concentrations are not reliable predictors of drug activity [27]. Instead, the area under the concentration‐time curve and gastric pH profile are better indicators [30]. We did not measure plasma drug levels in our current study, and the dosing regimen followed validated protocols from previous studies in healthy dogs [10, 31]. PPI therapy might have influenced pH‐based sampling; however, the esophageal microbiome (EM) obtained in this study differed substantially from the previously reported gastric microbiome in dogs [10]. This distinct difference increases the likelihood that our sampling accurately captured the esophageal microbiome.

16S‐rRNA amplicon sequencing was utilized in this study as a rapid and cost‐effective method for bacterial profiling of the EM in dogs [32]. However, next‐generation sequencing methods have been shown to have analytical variability and bias between protocols, bioinformatic pipelines, and laboratories, which could influence microbiome sequencing results and their comparisons [33, 34]. Reproducibility of results is affected due to the lack of a gold standard microbiome study design and systemic biases introduced due to methodological differences [35]. In addition, samples with lower DNA concentration have been shown to have increased technical variations across sequencing runs compared to samples with increased biomass [36]. Our study utilized one of the available sequencing methods and analytical pipelines, but due to the inherent variation and lack of validation between methods, additional studies might be required until a consensus is formed regarding the influence of short‐term omeprazole on the esophageal microbiome in dogs.

## 5 Conclusion

The EST was successfully used and well‐tolerated as a minimally invasive esophageal sampling device to investigate the EM in dogs. No significant changes were noted in alpha and beta diversities in the EM after short‐term omeprazole therapy in healthy dogs. The EST method offers a valuable foundation for studying EM alterations in dogs with esophageal diseases, as well as those at higher risk for aspiration and anesthetic complications.

## Disclosure

Authors declare no off‐label use of antimicrobials.

## Ethics Statement

Approved by the Institutional Animal Care and Use Committee at the Washington State University (protocol ASAF #7160).

## Conflicts of Interest

The authors declare no conflicts of interest.

## Supporting information


**Figure S1.** Esophageal string test kit, sampling procedure and retrieval (A) Esophageal string test kit (EnteroTrack LLC, Aurora, CO) containing a thermoplastic strip, a pair of scissors, forceps, measuring tape, and pH indicator. (B) EnteroTracker weighted capsule‐string technology; containing 90 cm of highly absorbent nylon string. (C) About 15–20 cm of string was pulled from the capsule until the thicker absorbent string was visible. (D) The capsule was administered orally via pilling. (E) The external portion of the string was secured to the dog’s collar using an adhesive tape to allow free head movement. (F) After 15 min, the string was retrieved by pulling it out of the mouth at an even rate (over 2–3 s).

**Figure S2.** Harvesting the esophageal portion of the string through pH‐based identification (A) pH marker used to differentiate the esophageal portion from gastric portion based on pH of collected biofluid (B) String displaying color difference using the pH indicator, with the left side indicating gastric biofluid and right indicating esophageal biofluid. (C) pH marker color chart (EnteroTrack LLC). (D) DNA/RNA shield (Zymo Research) collection tube to stabilize nucleic acid from specimens.

**Figure S3.** This figure represents the relative abundance of the taxa (expressed at the genus level) obtained from the string of the EST kit as a negative control. The taxa obtained are different from those of the esophageal biofluid using the EST.

**Figure S4.** This represents a bar plot showing the distribution of taxa at the phylum level in the esophageal biofluid of the test participant collected after 15 min of EST placement. The data demonstrates that a duration of 15 min obtained sufficient biofluid for microbiome analysis.

**Table S1.** Table representing the relative abundances of most abundant ASVs across all samples.

**Table S2.** Table containing the shared sequences (at least 50%) between all dogs across all time points.
