# Supplementary figures and images for: Effect of Omeprazole on Esophageal Microbiota in Dogs Detected Using a Minimally Invasive Sampling Method
Source: J Vet Intern Med. 2025 Feb 26;39(2):e70029. doi: 10.1111/jvim.70029 (PMC11864821; doi:10.1111/jvim.70029)

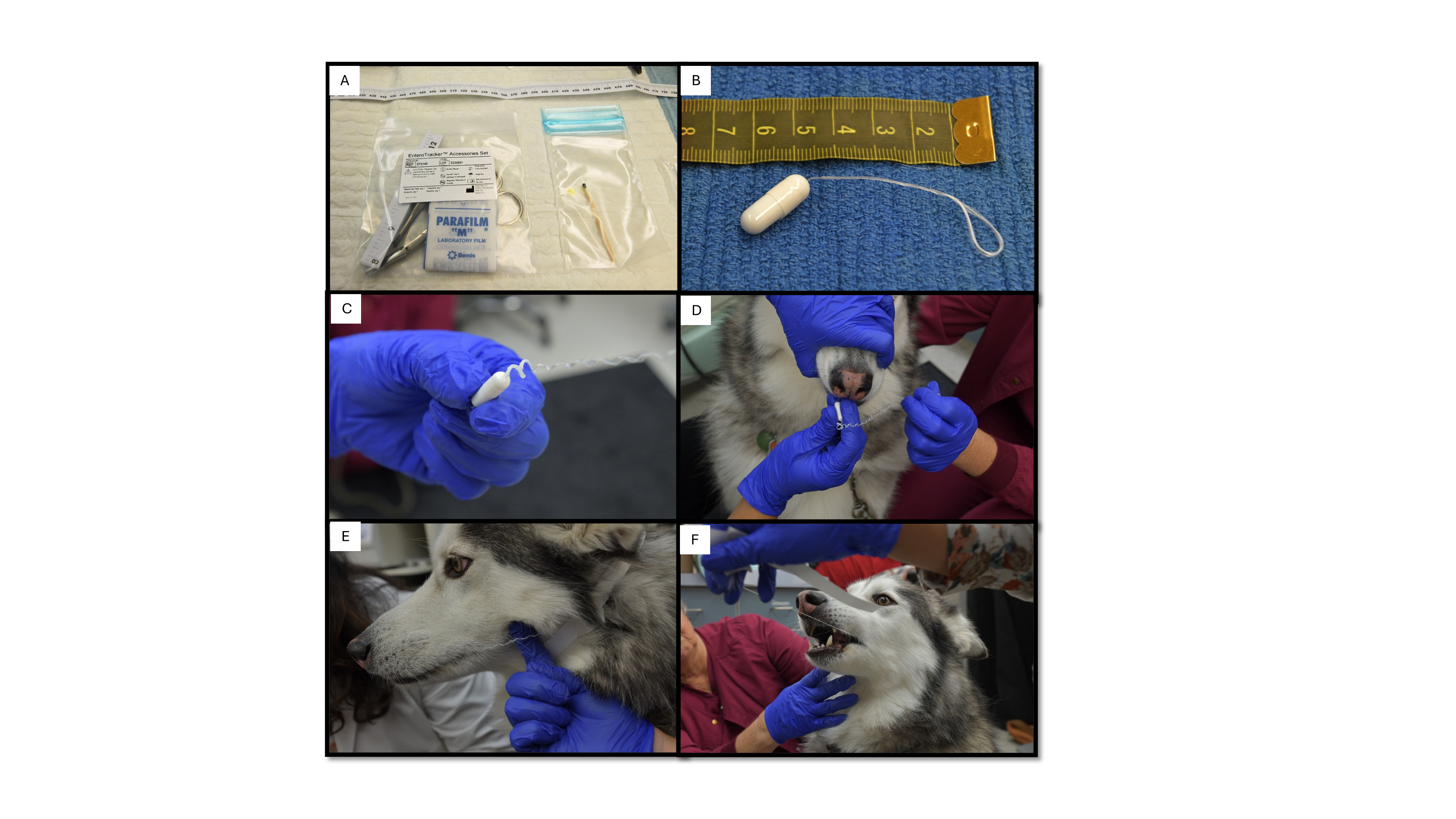

Supplement: Supplementary file 1 — Figure S1. Esophageal string test kit, sampling procedure and retrieval (A) Esophageal string test kit (EnteroTrack LLC, Aurora, CO) containing a thermoplastic strip, a pair of scissors, forceps, measuring tape, and pH indicator. (B) EnteroTracker weighted capsule‐string technology; containing 90 cm of highly absorbent nylon string. (C) About 15–20 cm of string was pulled from the capsule until the thicker absorbent string was visible. (D) The capsule was administered orally via pilling. (E) The external portion of the string was secured to the dog’s collar using an adhesive tape to allow free head movement. (F) After 15 min, the string was retrieved by pulling it out of the mouth at an even rate (over 2–3 s). [file JVIM-39-e70029-s002.tif]

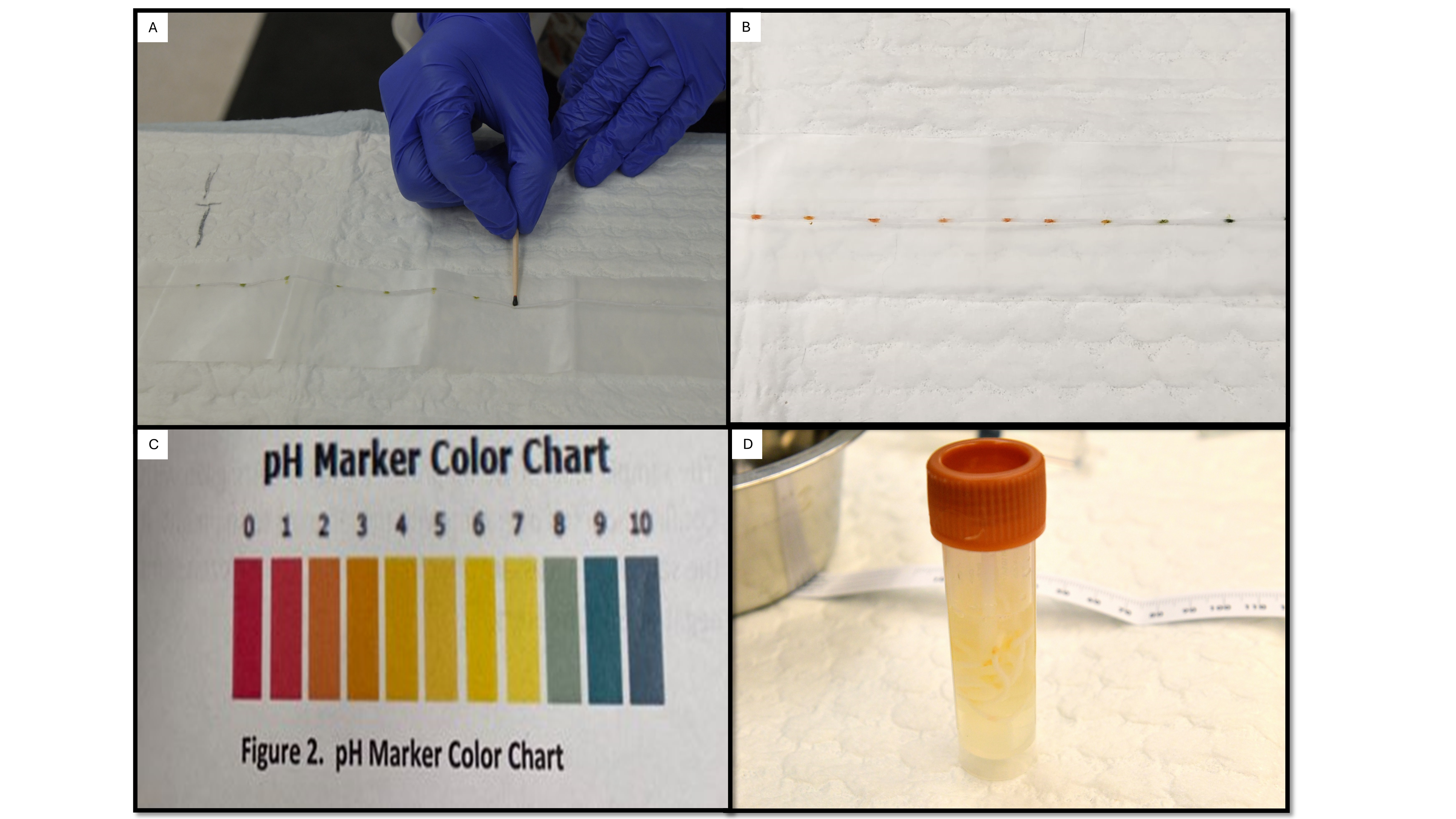

Supplement: Supplementary file 2 — Figure S2. Harvesting the esophageal portion of the string through pH‐based identification (A) pH marker used to differentiate the esophageal portion from gastric portion based on pH of collected biofluid (B) String displaying color difference using the pH indicator, with the left side indicating gastric biofluid and right indicating esophageal biofluid. (C) pH marker color chart (EnteroTrack LLC). (D) DNA/RNA shield (Zymo Research) collection tube to stabilize nucleic acid from specimens. [file JVIM-39-e70029-s003.tif]

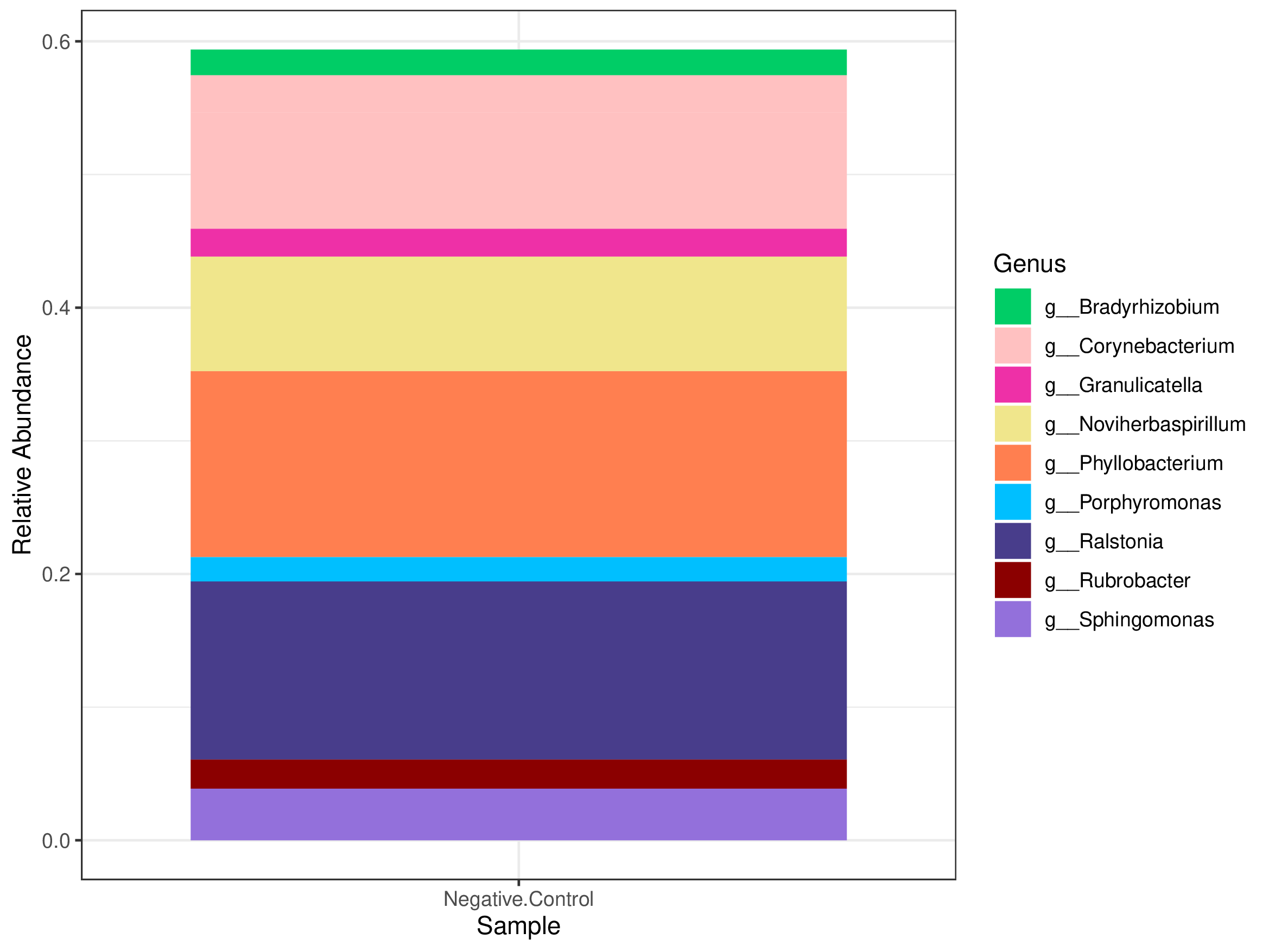

Supplement: Supplementary file 3 — Figure S3. This figure represents the relative abundance of the taxa (expressed at the genus level) obtained from the string of the EST kit as a negative control. The taxa obtained are different from those of the esophageal biofluid using the EST. [file JVIM-39-e70029-s006.tiff]

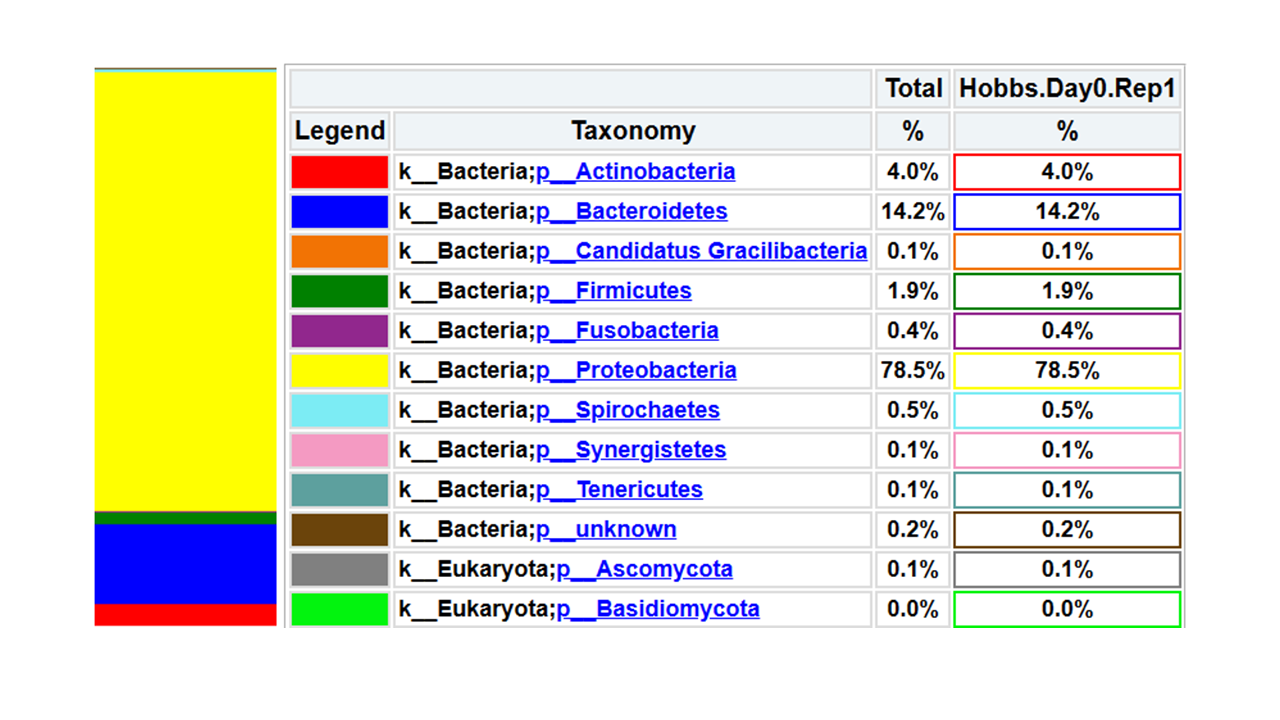

Supplement: Supplementary file 4 — Figure S4. This represents a bar plot showing the distribution of taxa at the phylum level in the esophageal biofluid of the test participant collected after 15 min of EST placement. The data demonstrates that a duration of 15 min obtained sufficient biofluid for microbiome analysis. [file JVIM-39-e70029-s005.tif]
